# Identification of the Six-RNA-Binding Protein Signature for Prognosis Prediction in Bladder Cancer

**DOI:** 10.3389/fgene.2020.00992

**Published:** 2020-08-28

**Authors:** Yucai Wu, Yi Liu, Anbang He, Bao Guan, Shiming He, Cuijian Zhang, Zhengjun Kang, Yanqing Gong, Xuesong Li, Liqun Zhou

**Affiliations:** ^1^Department of Urology, Peking University First Hospital, Beijing, China; ^2^Institute of Urology, Peking University, Beijing, China; ^3^National Urological Cancer Center, Beijing, China; ^4^Urogenital Diseases (Male) Molecular Diagnosis and Treatment Center, Peking University, Beijing, China; ^5^Department of Urology, The Fifth Affiliated Hospital of Zhengzhou University, Zhengzhou, China

**Keywords:** bladder cancer, RNA-binding proteins, overall survival, recurrence-free survival, prognosis

## Abstract

RNA-binding proteins (RBPs) are a kind of gene regulatory factor that presents a significant biological effect in the initiation and development of various tumors, including bladder cancer (BLCA). However, the RBP-based prognosis signature for BLCA has not been investigated. In this study, we attempted to develop an RBP-based classifier to predict overall survival (OS) for BLCA based on transcriptome analysis. We extracted data of BLCA patients from The Cancer Genome Atlas database (TCGA) and UCSC Xena. Finally, a total of 398 cases without missing clinical data were enrolled and six RBPs (*FLNA*, *HSPG2*, *AHNAK*, *FASTKD3*, *POU5F1*, and *PCSK9*) associated with OS of BLCA were identified through univariate and multivariate Cox regression analysis. Online analyses and immunohistochemistry validated the prognostic value and expression of six RBPs. Risk scores were calculated to divide patients into high-risk and low-risk level, and patients in the high-risk group tended to have a poor prognosis. In addition, the receiver operating characteristic (ROC) curve analysis was performed to assess the prognostic value of RBPs, and the area under the curve (AUC) values were 0.711 and 0.706, respectively, in the training set and validating set. The findings were further validated in an external validation set. Subsequently, the 6-RBP-based signature and pathological stage were used to construct the nomogram to predict the 3- and 5-years OS of BLCA patients. Also, this 6-RBP-based signature was highly related to recurrence-free survival of BLCA. Weighted co-expression network analysis (WGCNA) combined with functional enrichment analysis contributed to study the potential pathways of six RBPs, including keratinocyte differentiation, RHO GTPases activate PNKs, epithelial tube morphogenesis, establishment or maintenance of cell polarity, and so on. In summary, the 6-RBP-based signature holds the potentiality to serve as a novel prognostic predictor of OS for BLCA.

## Introduction

Bladder cancer (BLCA) is the 10th most prevalent cancer and the most frequently diagnosed malignancy of the urinary system all over the world (Bray et al., 2018). It has been estimated that there will be 81,440 cases of newly diagnosed BLCA and 17,980 people will die for BLCA in 2020 in the United States (Siegel et al., 2020). Non-metastatic BLCA is separated into non-muscle-invasive bladder cancer (NMIBC) and muscle-invasive bladder cancer (MIBC) and approximately 70% of BLCA patients belong to NMIBC when initially diagnosed (Dobruch et al., 2016). MIBC patients have a more favorable prognosis than those with locally advanced and metastatic BLCA due to the limited effects of surgery on advanced BLCA. In addition, BLCA is the cancer with high recurrence and about half of patients after radical surgery relapse and present with metastases (Alfred Witjes et al., 2017). However, no specific symptoms appeared in the early stage of tumor, which makes it urgent to develop novel biomarkers to predict the survival of BLCA.

RNA-binding proteins (RBPs) are a kind of key factors regulating the process of tumorigenesis, and each step that led to the initiation of malignancy may involve one or more RBPs. Mechanisms of RBPs regulation have been identified in cancer cells, including alternative splicing, polyadenylation, stability, subcellular localization, and translation (Pereira et al., 2017). Post-transcriptional regulation is an essential way of promoting or suppressing oncogenesis. RBPs can interact with other proteins and coding or non-coding RNA to form the ribonucleoprotein complexes. For example, RBPs can interact with microRNAs (miRNAs) (Liang et al., 2020), long non-coding RNAs (lncRNAs) (Jiang et al., 2020), and circular RNAs (circRNAs) (Chen et al., 2019) to affect tumor progression.

Initial assessment of BLCA has been explored in recent times. In clinical practice, lncRNAs, miRNAs, and clinicopathological factors including TNM stage and lymph node status have been gradually used to assess BLCA prognosis. Recently increasing researches have revealed that RBPs were associated with the prognosis of patients (Busa et al., 2007; Vo et al., 2012). Therefore, we aimed to identify a number of RBPs as potential biomarkers based on transcriptome analysis to predict the outcome of BLCA. We constructed a 6-RBP-based classifier for OS by using the multivariable Cox regression, which could optimize the predictivity of the current TNM staging system. Patients with gene sequencing data from the GSE13507 database were adopted as the external validation. In addition, this 6-RBP-based classifier was also highly relevant to recurrence-free survival (RFS) in BLCA. Our results demonstrated that the 6-RBP-based classifier could be used as reliable prognostic predictors of BLCA survival and recurrence.

## Materials and Methods

### Data Acquisition

The TCGA database was used to obtain transcriptome profiling data of tumor and normal tissues. Then, 19 normal samples and 411 BLCA samples were obtained. The matrix of mRNA expression was extracted separately by annotations using Gencode (GENCODE v 26) GTF file. Those genes whose expression was “zero” in 90% of BLCA patients were eliminated. Clinical data were downloaded from the UCSC Xena website^1^. To analyze the correlation of gene expression signatures with the OS of BLCA patients, we filtered out samples without clinical survival information; then, we selected a total of 398 patients and these patients were divided into training (*n* = 265) and validating set (*n* = 133) randomly at a 2:1 ratio for further analysis. Microarray study and its clinical information (GSE13507) in Gene Expression Omnibus (GEO) database^2^ (*n* = 165) were extracted, profiled by the Illumina human-6 v2.0 expression BeadChip platform. A total of 1348 genes coding for RBPs including those with high confidence for RNA binding and those annotated as RNA binding in Ensembl were summarized from the published literature (Baltz et al., 2012; Castello et al., 2012; Kwon et al., 2013; Cunningham et al., 2015).

### Analysis of Differentially Expressed Genes

We used the R package edgeR to obtain differentially expressed genes (DEGs), where | log2 fold change (FC)| >1, *P* <0.05, and false discovery rate (FDR) <0.05 were used as the cutoffs. Then, we filtered the DEGs coding for RNA-binding proteins (DERBPs). R package “heatmap” was performed to display the selected six DERBPs.

### Data Processing and Risk Score Calculation

The DERBPs were subjected to univariable Cox regression analysis to select DERBPs that were associated with OS of BLCA patients. We selected those DERBPs with *P* <0.001 into multivariable Cox regression to obtain the coefficients. Then, six DERBPs significantly correlated with OS were identified to build up the prediction model weighted by their coefficients. A risk score formula for OS was constructed, and each patient had been assigned a risk score according to this risk score formula that was a linear combination of the expression levels of the significant DERBPs weighted by their respective Cox regression coefficients. Then, we divided patients into low-risk group and high-risk group according to the median risk score.

### Weighted Co-expression Network Analysis (WGCNA)

Considering our risk score model was built based on the expression of six RBPs, we constructed a weight co-expression network of risk score gene with DEGs in BLCA to explore the biological function by the R package “WGCNA” (Langfelder and Horvath, 2008). We selected 3 as the soft thresholding power to produce a scale-free network and the enrolled genes were hierarchically clustered into 16 modules, where the red module was found to be most relevant to risk score.

### Pathway Enrichment Analysis

In order to explore the potential functions of the 6-RBP signature, genes in the red module were picked up for enrichment analysis. Pathway enrichment was conducted using an online web tool “Metascape^3^.” The significance threshold of FDR for significantly enriched biological processes and pathways was set at 0.05.

### Statistical Analysis

We use Chi-squared test or Fisher’s exact test to measure the difference between training and validating sets and the relationship between clinical data and risk score. Both univariable and multivariable Cox regression analysis were performed using the R package “survival.” The Kaplan–Meier survival curve was drawn to demonstrate the relationship between DERBPs and OS or RFS. The log-rank test was constructed to test the significance of the difference between the two groups. ROC analysis was performed to measure prognostic accuracy. All statistical tests were two-sided, and *P* <0.05 was considered statistically significant. All analyses were performed in SPSS version 25.0 (SPSS Inc., Chicago, IL, United States) or R version 3.5.2^4^ with the following packages: “edgeR” (Robinson et al., 2010), “glmnet,” “gplot,” and “survivalROC.”

## Results

### Data Source and Processing

**FIGURE 1:**
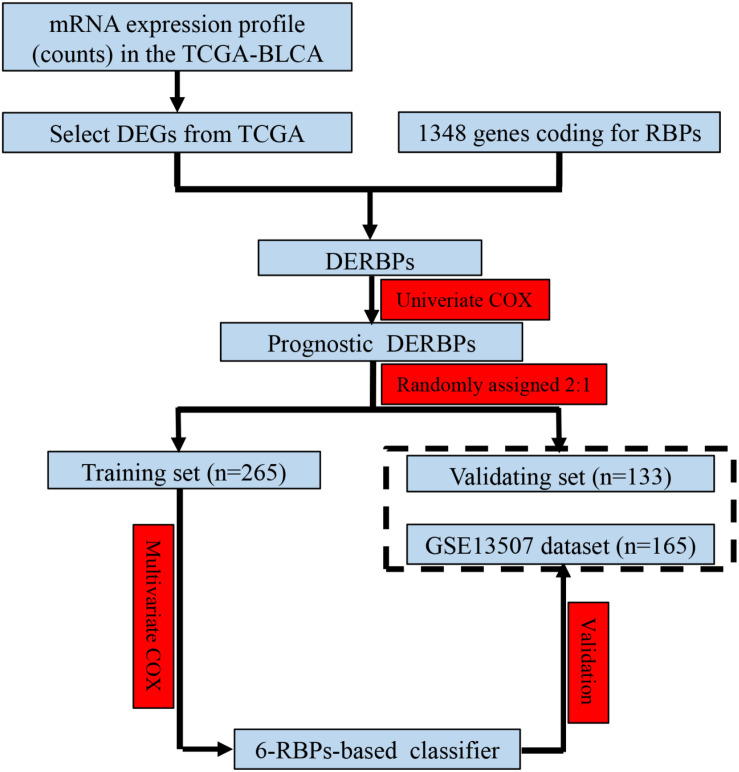
Study flowchart showing the process of constructing the 6-RBP-based signature to predict overall survival (OS) of bladder cancer (BLCA).

**FIGURE 2:**
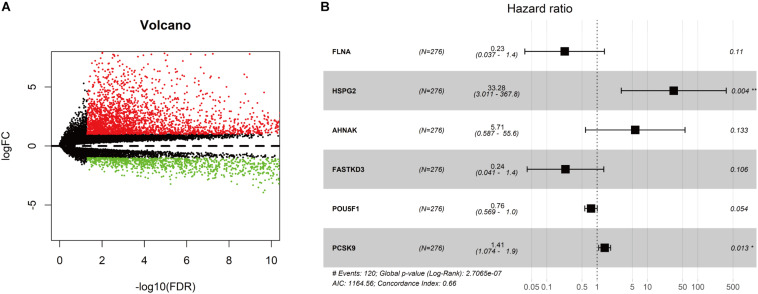
Screening for differentially expressed genes in bladder cancer (BLCA). **(A)** Volcano plot of differentially expressed genes in the TCGA-BLCA cohort. The red dot represented the upregulated gene, while the green dot represented the downregulated gene. **(B)** Forest plot exhibited the hazard ratios (HRs) with 95% confidence interval (95% CI) of prognostic RBPs in BLCA on the basis of the multivariate Cox regression result.

As shown in Figure 1, we obtained 19 normal samples and 411 BLCA samples from TCGA database, and 4456 DEGs with | Log2FC| >1 and FDR <0.05 were identified using edgeR (Figure 2A). A total of 1348 genes coding known or predicted RBPs were matched with the 4456 DEGs and then 109 RBPs remained. After that, univariate Cox regression was performed to choose factors to predict prognostic of patients and 12 DERBPs with *P* <0.001 were retained for further analysis. Clinical characters of BLCA patients were downloaded from the UCSC database, and these cases were randomly divided into training set (*n* = 265) and validating set (*n* = 133) at a 2:1 ratio. There were no significant differences in age, gender, pathological stage, histologic grade, and diagnosis subtype between two sets (Table 1). Then, we identified six DERBPs, which were strongly associated with OS of BLCA by multivariate Cox regression analysis in the training set (Figure 2B), and the detailed information of these RBPs including *FLNA (Filamin A)*, *HSPG2 (Heparan Sulfate Proteoglycan 2)*, *AHNAK (AHNAK Nucleoprotein)*, *FASTKD3 (FAST Kinase Domains 3)*, *POU5F1 (POU Class 5 Homeobox 1)*, and *PCSK9 (Proprotein Convertase Subtilisin/Kexin Type 9)* were listed in Table 2. Among these genes, higher expression of *HSPG2*, *AHNAK*, and *PCSK9* was associated with decreased survival. On the contrary, higher expression of *FLNA*, *FASTKD3*, and *POU5F1* was related to increased survival.

**TABLE 1:** Clinical features of BLCA patients in the training and validating sets.

Features	Training set (*n* = 265)	Validating set (*n* = 133)	Pearson χ^2^	*P*
**Age (years), *n* (%)**				
≤70	138 (52.1)	72 (54.1)		
>70	127 (47.9)	61 (45.9)	0.151	0.698
**Gender, *n* (%)**				
Male	189 (71.3)	100 (75.2)		
Female	76 (28.7)	33 (24.8)	0.666	0.414
**Pathological stage, *n* (%)**				
I + II	85 (32.1)	41 (30.8)		
III + IV	180 (67.9)	92 (69.2)	0.064	0.801
**Histologic grade, *n* (%)**				
Low	13 (4.9)	7 (5.3)		
High	252 (95.1)	126 (94.7)	0.024	0.878
**Diagnosis subtype, *n* (%)**				
Non-papillary	175 (66.0)	96 (72.2)		
Papillary	90 (34.0)	37 (27.8)	1.538	0.215

*BLCA, Bladder cancer. Bold values are significant to p < 0.05.*

**TABLE 2:** Six RBPs significantly associated with the OS of BLCA patients in the training set.

Gene symbol	ENSG ID	Coefficient	*P*	HR	HR (95% CI)
FLNA	ENSG00000196924	−1.48536	0.11	0.23	0.037–1.4
HSPG2	ENSG00000142798	3.504967	**0.004**	33.28	3.011–367.8
AHNAK	ENSG00000124942	1.743093	0.133	5.71	0.587–55.6
FASTKD3	ENSG00000124279	−1.44735	0.106	0.24	0.041–1.4
POU5F1	ENSG00000204531	−0.27992	**0.054**	0.76	0.569–1.0
PCSK9	ENSG00000169174	0.346457	**0.013**	1.41	1.074–1.9

*BLCA, Bladder cancer; OS, Overall survival; HR, Hazard ratio; 95% CI, 95% Confidence interval. Bold values are significant to p < 0.05.*

### Validation the Prognostic Value and Expression of Six RBPs

**FIGURE 3:**
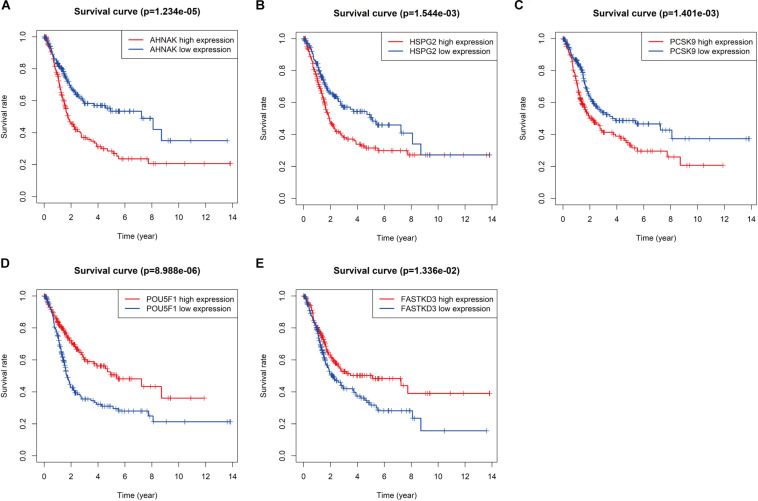
Kaplan–Meier analysis for overall survival (OS) of bladder cancer (BLCA) patients based on the mRNA expression of *AHNAK* **(A)**, *HSPG2* **(B)**, *PCSK9* **(C)**, *POU5F1* **(D)**, and *FASTKD3* **(E)**.

**FIGURE 4:**
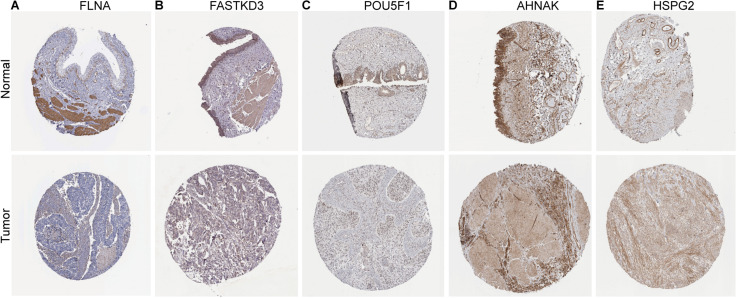
Validation of the expression of RNA-binding proteins (RBPs) in bladder cancer (BLCA) and normal tissues in the Human Protein Atlas (HPA) database. **(A)** *FLNA*, **(B)** *FASTKD3*, **(C)** *POU5F1*, **(D)** *AHNAK*, and **(E)** *HSPG2*.

To further explore the prognostic value of six RBPs in BLCA, the Kaplan–Meier plotter was used to determine the relationship between six RBPs and OS. Five of the six RBPs *(AHNAK*, *HSPG2*, *PCSK9*, *POU5F1*, and *FASTKD3*) were identified. Results of log-rank test demonstrated that the high expressions of *AHNAK*, *HSPG2*, and *PCSK9* were associated with the low OS, while the high expression of *POU5F1* and *FASTKD3* was associated with the high OS of BLCA patients (Figure 3). To further validate the expression of these RBPs in BLCA, we analyzed immunohistochemistry data from the Human Protein Atlas (HPA) database^5^ to show that *FLNA*, *FASTKD3*, and *POU5F1* were significantly decreased in BLCA compared with normal urinary bladder tissue (Figures 4A–C). Besides, the staining level of *HSPG2* was increased in BLCA (Figure 4E). However, the staining level of *AHNAK* was relatively reduced in normal urinary bladder tissue and the result of *PCSK9* protein expression was not detectable (Figure 4D). These results showed that expression of each of the six RBPs was related to prognosis of BLCA.

### Development and Validation a 6-RBP-Based Classifier to Predict OS of BLCA

**FIGURE 5:**
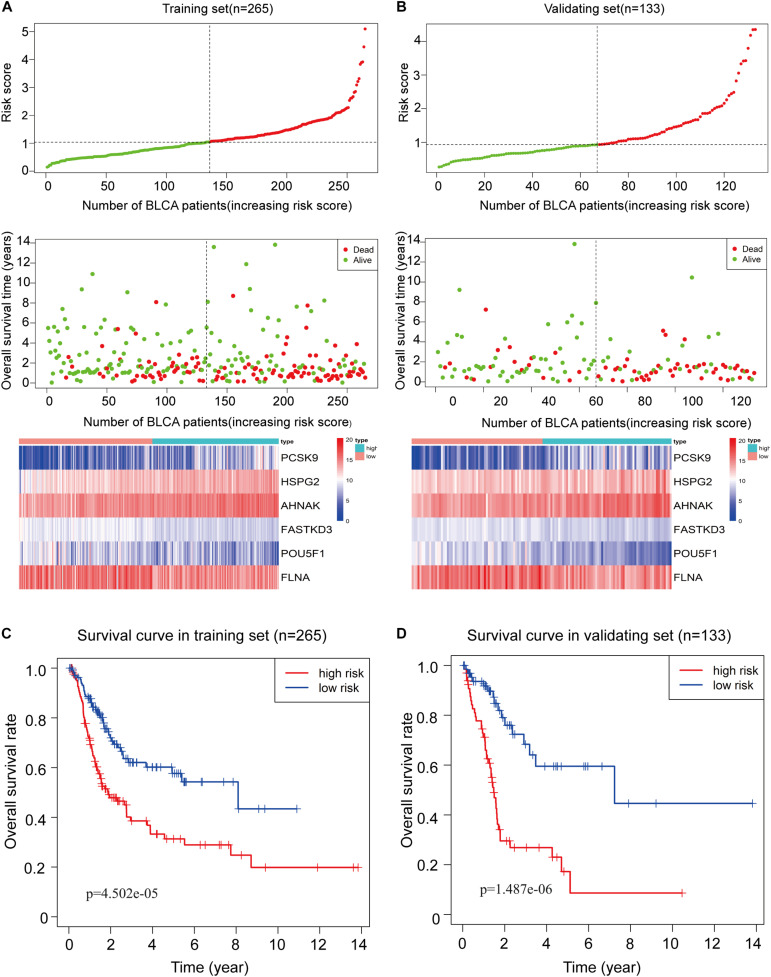
Construction of the 6-RBP-based risk model of bladder cancer (BLCA). **(A)** The 6-RBP-based risk score distribution, living status of BLCA patients, and heatmap of six RBP expression profiles in the training set. **(B)** The 6-RBP-based risk score distribution, living status of BLCA patients, and heatmap of six RBP expression profiles in the validating set. **(C,D)** Kaplan–Meier analysis for overall survival (OS) of BLCA patients based on the risk stratification in the training set **(C)** and validating set **(D)**.

To assess the ability of the 6-RBP-based model predicting survival of BLCA, we created a risk score according to the expression of six RBPs as follows: Risk score = (3.50 ^∗^ expression value of *HSPG2*) + (1.74 ^∗^ expression value of *AHNAK*) + (0.35 ^∗^ expression value of *PCSK9*) - (1.49 ^∗^ expression value of *FLNA*) - (1.45 ^∗^ expression value of *FASTKD3*) - (0.28 ^∗^ expression value of *POU5F1*). Then, we calculated risk score according to this formula and cases were divided into high-risk and low-risk group based on the cutoff of median risk score (Figure 5A). The mortality was higher in the high-risk group than that in the low-risk group [HR: 2.274 (95% CI: 1.562–3.312), *p* < 0.001]. Moreover, *HSPG2*, *AHNAK*, and *PCSK9* were highly expressed in the high-risk group, while *FLNA*, *FASTKD3*, and *POU5F1* were highly expressed in the low-risk group. Results in the validating set were consistent with findings described above (Figure 5B). Kaplan–Meier curves revealed that patients in the high-risk group had shorter OS than those in the low-risk group (*p* < 0.001) in the training set (Figure 5C), and this result was further confirmed in the validating set (*p* < 0.001) (Figure 5D).

**FIGURE 6:**
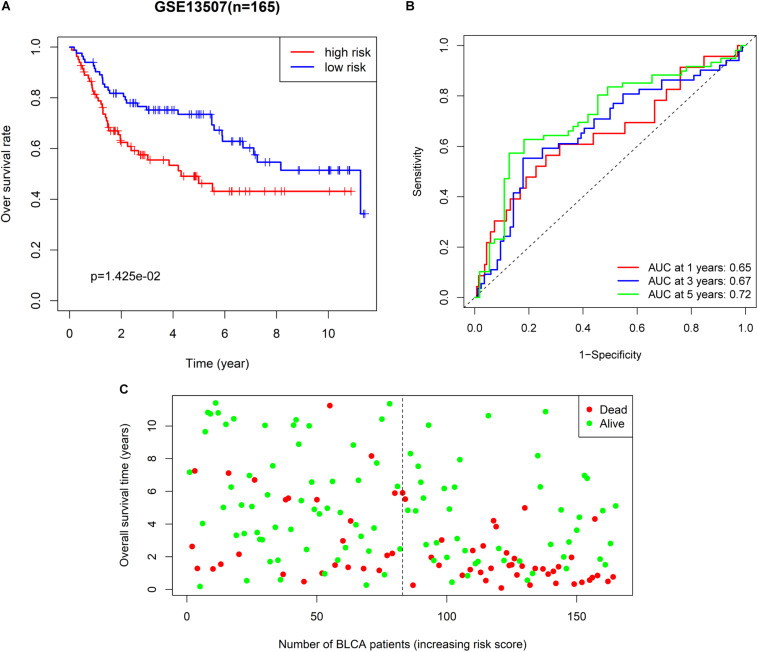
Further validation of the 6-RBP-based risk model in the GSE13507 dataset. **(A)** Kaplan–Meier analysis for overall survival (OS) of bladder cancer (BLCA) patients based on the risk stratification. **(B)** Receiver operating characteristic (ROC) analysis for OS prediction including 1–, 3–, and 5–years of BLCA patients. **(C)** The living status of BLCA patients.

We also extracted BLCA samples from the GSE31507 database (*n* = 165) to validate the ability of the 6-RBP-based classifier predicting OS of BLCA. Results were compatible with that in the training and validating set derived from TCGA database. Patients in the high-risk group had shorter OS than those in the low-risk group (*p* = 0.014) (Figure 6A). We also found that our 6-RBP-based classifier performed well in predicting 5-year OS of BLCA (AUCs = 0.721) (Figure 6B). The mortality was higher in the high-risk group than that in the low-risk group (Figure 6C).

### Correlation Between RBPs Classifier and Clinicopathologic Characteristics

**FIGURE 7:**
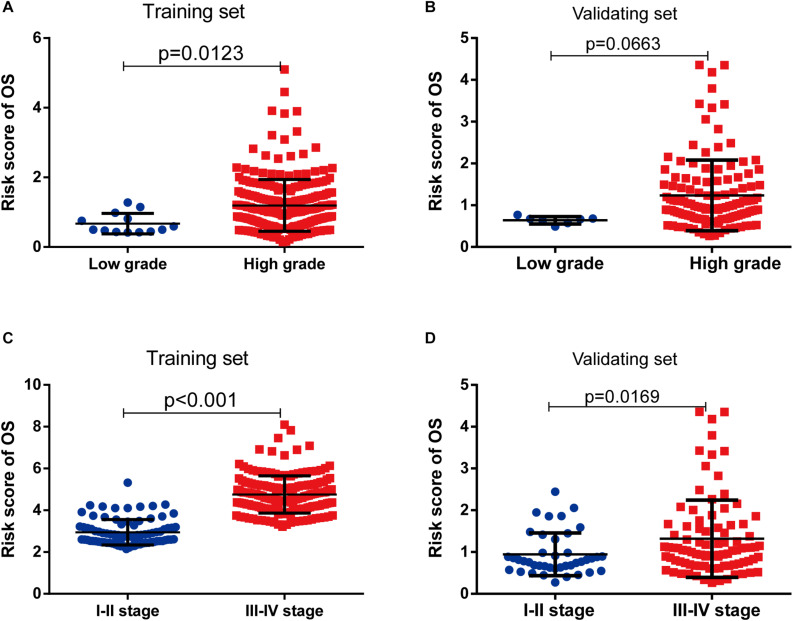
The risk score was associated with histological grade and pathologic stage of bladder cancer (BLCA). **(A,B)** Boxplot of risk score in patients with different histological grade in the training set **(A)** and validating set **(B)**. **(C,D)** Boxplot of risk score in patients with different pathologic stage in the training set **(C)** and validating set **(D)**.

As shown in Table 3, clinicopathologic characteristics age (*p* = 0.032), pathological stage (*p* < 0.001), histologic grade (*p* = 0.011), and diagnosis subtype (*p* = 0.002) showed significant differences between the high-risk group and low-risk group in the training set. Only age (*p* = 0.046), pathological stage (*p* = 0.002), and histologic grade (*p* = 0.007) displayed distinct differences in the validating set. Patients with high pathological stage and histologic grade were prone to get a high-risk score in two sets (Figure 7).

**TABLE 3:** Clinicopathological characteristics of the 6-marker-based classifier with OS in the training set and validating set.

Features	Training set (*n* = 265)			Validating set (*n* = 133)		
	Low risk (*n* = 133)	High risk (*n* = 132)	*P*	Low risk (*n* = 67)	High risk (*n* = 66)	*P*
**Age (years), *n* (%)**						
≤70	78 (29.4)	60 (22.6)		42 (31.6)	30 (22.6)	
>70	55 (20.8)	72 (27.2)	**0.032**	25 (18.8)	36 (27.1)	**0.046**
**Gender, *n* (%)**						
Male	101 (38.1)	88 (33.2)		52 (39.1)	48 (36.1)	
Female	32 (12.1)	44 (16.6)	0.095	15 (11.3)	18 (13.5)	0.514
**Pathological stage, *n* (%)**						
I + II	58 (21.9)	27 (10.2)		29 (21.8)	12 (9.0)	
III + IV	75 (28.3)	105 (39.6)	**<0.001**	38 (28.6)	54 (40.6)	**0.002**
**Histologic grade, *n* (%)**						
Low	11 (4.2)	2 (0.8)		7 (5.3)	0 (0)	
High	122 (46.0)	130 (49.1)	**0.011**	60 (45.1)	66 (49.6)	**0.007**
**Diagnosis subtype, *n* (%)**						
Non-papillary	76 (28.7)	99 (37.4)		45 (33.8)	51 (38.3)	
Papillary	57 (21.5)	33 (12.5)	**0.002**	22 (16.5)	15 (11.3)	0.193

*OS, Overall survival. Bold values are significant to p < 0.05.*

### Prognostic Value of RBPs Classifier for Assessing Overall Survival

The 6-RBP-based classifier, age, and pathological stage were significantly related to the OS in the univariate Cox regression analysis. After the multivariate Cox regression analysis of the abovementioned factors, the 6-RBP-based classifier model and pathological stage were retained to be dependable factors for OS in the training set. Except for age, similar results were observed in the validating set (Table 4). Our result showed that the 6-RBP-based classifier was an independent prognostic factor for OS in BLCA in two sets.

**TABLE 4:** Univariate and multivariate Cox regression analysis of the 6-marker-based classifier with OS in the training set and validating set.

Features	Univariate COX		Multivariate COX	
	HR (95% CI)	*P*	HR (95% CI)	*P*
**Training set**				
Age (>70 vs. ≤70)	1.519 (1.049, 2.200)	**0.027**		
Gender (Male vs. Female)	0.831 (0.562, 1.231)	0.356		
Pathological stage (III + IV vs. I + II)	2.210 (1.387, 3.521)	**0.001**	1.826 (1.135, 2.937)	**0.013**
Histologic grade (High vs. Low)	1.942 (0.477, 7.899)	0.354		
Diagnosis subtype (Papillary vs. Non-papillary)	0.674 (0.441, 1.028)	0.067		
6-marker-based classifier (High risk vs. Low risk)	1.738 (1.435, 2.103)	**<0.001**	1.636 (1.342, 1.995)	**<0.001**
**Validating set**				
Age (>70 vs. ≤70)	1.853 (1.106, 3.105)	**0.019**	1.861 (1.084, 3.195)	**0.024**
Gender (Male vs. Female)	0.916 (0.508, 1.649)	0.769		
Pathological stage (III + IV vs. I + II)	2.573 (1.301, 5.089)	**0.007**	2.497 (1.234, 5.052)	**0.011**
Histologic grade (High vs. Low)	1.278 (0.019, 8.561)	0.394		
Diagnosis subtype (Papillary vs. Non-papillary)	0.571 (0.280, 1.163)	0.123		
6-marker-based classifier (High risk vs. Low risk)	3.781 (2.117, 6.754)	**<0.001**	3.166 (1.751, 5.727)	**<0.001**

*OS, Overall survival; HR, Hazard ratio; 95% CI, 95% Confidence interval. Bold values are significant to p < 0.05.*

**FIGURE 8:**
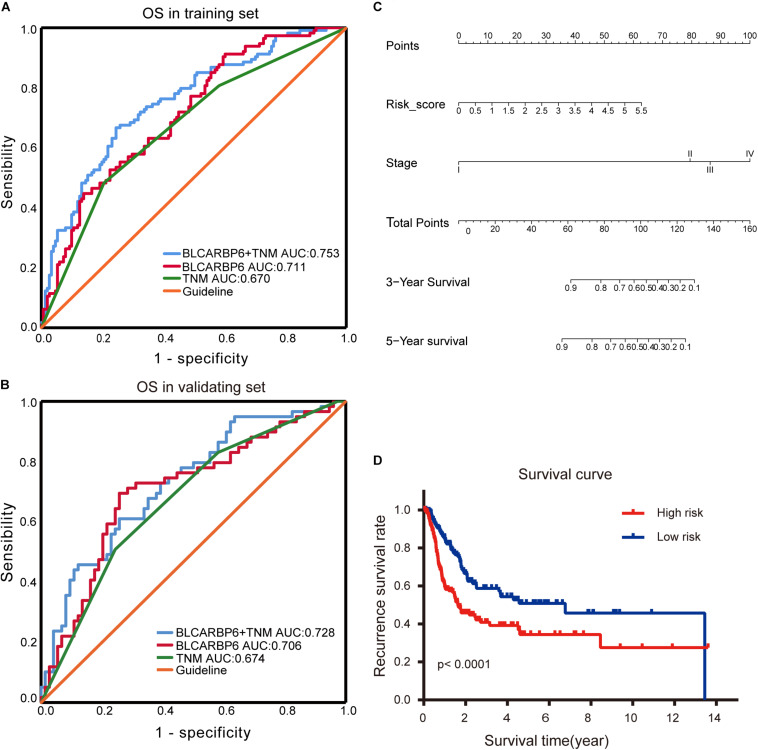
Estimate the predictive ability of RBP-based classifier to predict survival in bladder cancer (BLCA) patients. **(A,B)** The receiver operating characteristic (ROC) for TNM stage only, the RBP-based risk score (BLCARBP6), and the RBP-based risk score combined with TNM stage for overall survival (OS) in the training set **(A)** and validating set **(B)**. **(C)** Nomogram to predict the 3- and 5-years OS. **(D)** Kaplan–Meier analysis for recurrence-free survival (RFS) of BLCA patients based on the risk stratification.

In order to evaluate the capabilities of the 6-RBP-based signature to predict OS of BLCA, we plotted the ROC curves and AUC was also calculated in both cohorts. AUC values in the training set and validating set were 0.711 and 0.706, respectively, which showed that the RBP-based classifier model had an obvious better predictive accuracy compared with the TNM staging (0.670 and 0.674, respectively, in the training set and validating set) (Figures 8A,B). In consideration of the role of TNM staging in clinical practice, we combined the RBP-based model and TNM staging to predict OS of BLCA. AUC values of this joint prediction model in the training and validating set were 0.753 and 0.728, indicating that this model was more accurate than models enrolled in RBPs or TNM staging solely. Subsequently, the 6-RBP-based risk score and pathological stage were used to construct the nomogram to predict the 3- and 5-year OS of BLCA patients (Figure 8C).

### Prognostic Value of the RBP-Based Classifier for Assessing RFS

We further explored whether this 6-RBP-based classifier was related to RFS of BLCA. As shown in Table 5, univariate and multivariate Cox regression analysis was conducted to identify prognostic factors for RFS. Finally, outcomes of univariate and multivariate analysis indicated that pathological stage and the 6-RBP-based classifier were independent risk factors of RFS in BLCA patients. Survival analysis revealed that the RFS of patients in the high-risk group were considerably shorter than that in the low-risk group (Figure 8D). Our results demonstrated that this 6-RBP-based classifier could also be used as a reliable prognostic predictor of BLCA recurrence.

**TABLE 5:** Univariate and multivariate Cox regression analysis of the 6-marker-based classifier with RFS in TCGA database.

Features	Univariate COX		Multivariate COX	
	HR (95% CI)	*P*	HR (95% CI)	*P*
Age (>70 vs. ≤70)	1.117 (0.802, 1.557)	0.513		
Gender (Male vs. Female)	0.857 (0.596, 1.232)	0.404		
Pathological stage (III + IV vs. I + II)	2.879 (1.810, 4.578)	**<0.001**	2.623 (1.643, 4.187)	**<0.001**
Histologic grade (High vs. Low)	3.345 (0.826, 13.544)	0.091		
Diagnosis subtype (Papillary vs. Non-papillary)	0.642 (0.431, 0.956)	**0.029**		
6-marker-based classifier (high risk vs. low risk)	1.911 (1.365, 2.677)	**<0.001**	1.696 (1.207, 2.382)	**0.002**

*RFS, Recurrence-free survival; TCGA, The Cancer Genome Atlas; HR, Hazard ratio; 95% CI, 95% Confidence interval. Bold values are significant to p < 0.05.*

### Pathway Enrichment Analysis of DERBPs

**FIGURE 9:**
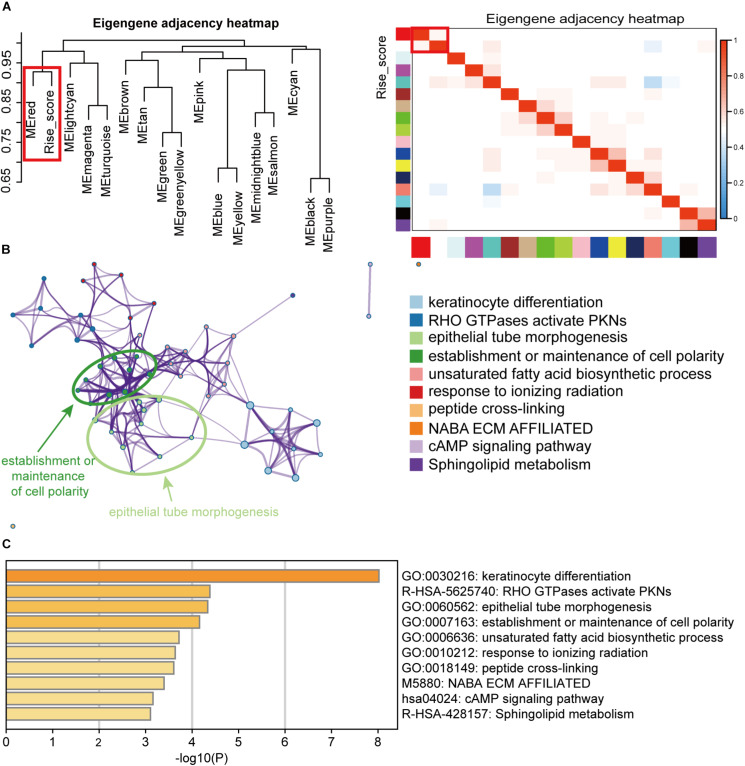
Pathway enrichment analysis of the RBPs signature. **(A)** Weighted co-expression network analysis (WGCNA) plot was performed to cluster genes associated with the 6-RBP-based risk score, and the data of the risk score were added to construct the eigengene adjacency heatmap. **(B)** Pathways associated with the 6-RBP-based signature were enriched by Metascape. **(C)** The histogram of the top 10 enriched pathways associated with the 6-RBP-based risk score. The abscissa was the value of -Log10P, and the longitudinal axis denotes different enrichment pathways, sorted by the value of -Log10P.

To explore the biological function of the 6-RBP signature in BLCA, the WGCNA method was performed to choose genes associated with risk score. Sixteen modules were identified by hierarchical clustering analysis, and the red module was significantly associated risk score (Figure 9A). In addition, genes in the red module served to perform pathway enrichment analysis. Results of enrichment analysis showed that genes were mostly enriched in keratinocyte differentiation, and RHO GTPases activate PNKs, epithelial tube morphogenesis, establishment or maintenance of cell polarity, and so on, suggesting that these pathways were correlated with the disease progression of BLCA with high-risk score (Figures 9B,C).

## Discussion

RBPs were regarded acting as amplifiers of oncogenic driver mutations. Increasing literature demonstrated that RBPs were of vital importance in the initiation, development, and recurrence of multi-malignancies. For instance, *LIN28B* overexpression promoted tumorigenesis and metastasis of colon cancer via repressing the level of let-7 microRNAs (King et al., 2011). RBPs also played a critical role in the initiation of BLCA. It was reported that *HuR* was upregulated in the BLCA tissue compared with adjacent normal tissue and it could promote BLCA progression by competitively binding to the long non-coding HOX transcript antisense RNA with miR-1 (Yu et al., 2017). RBPs also exhibit potentiality as novel biomarkers. Pancreatic ductal adenocarcinoma patients with higher levels of *ESRP1* showed longer survival than those with low *ESRP1* expression (Ueda et al., 2014). In conclusion, RBPs can regulate the biology of cancer and hold potential as novel biomarkers.

Diverse models to predict the outcome of BLCA have been created, including miRNA-based signature (Hofbauer et al., 2018; Yin et al., 2019), clinical character-based nomogram (Zhang et al., 2019), and lncRNA-based model (Wang et al., 2020). Parts of them performed well in predicting the survival of BLCA. However, no RBP-based classifier for predicting survival in BLCA has been established. RBPs are a subset molecule of regulating progression and development of malignancies. Considering the limited capabilities of single RBP for prediction prognosis, we constructed a predicted model based on mRNA expression of six RBPs by univariate and multivariate Cox regression analysis. Patients were divided into two categories based on the median-risk score and high-risk patients have shorter survival than low-risk patients, suggesting that our model displayed powerful strength to forecast the OS for BLCA. Clinicopathologic characteristics including age, pathological stage, and histologic grade were associated with the expression of six RBPs and patients with high stage or grade tended to get high-risk score. Our 6-RBP-based classifier with AUCs being 0.711 and 0.706 in the training and validating sets presented a strong ability to predict OS of BLCA. In addition, TNM stage is the most commonly used index to assess the treatment way and outcome of BLCA. We also combined the 6-RBP-based signature together with TNM stage to assess the prognosis and the AUCs showed that this model was more accurate than the 6-RBP-based model. Furthermore, we explored the efficiency of the 6-RBP-based classifier in predicting RFS of BLCA. Results showed that pathological stage and the 6-RBP-based classifier were independent factors of RFS in BLCA patients and patients in the low-risk group showed a significantly longer RFS than those in the high-risk group.

Six prognosis-related RBPs including *FLNA*, *HSPG2*, *AHNAK*, *FASTKD3*, *POU5F1*, and *PCSK9* were selected to build a classifier. It has been confirmed that *FLNA* was downregulated in BLCA tissue and overexpression of *FLNA* repressed migration, invasion, and migration of BLCA by regulating autophagy (Wang et al., 2018). *AHNAK* downregulated in BLCA performed accurately on discriminating between benign urothelial lesion and bladder urothelial carcinoma using voided-urine liquid-based cytology (Lee et al., 2018). In addition, low expression of *POU5F1* was associated with shorter cancer-related survival and might be a novel biomarker for BLCA (Chang et al., 2008). However, functions of *HSPG2*, *FASTKD3*, and *PCSK9* in BLCA have not been explored yet. These confirmed or predicted prognosis-related RBPs support evidence that our model is capable of assessing the outcome of BLCA.

In order to explore the biological function of the 6-RBP signature, we performed WGCNA and pathway enrichment analysis. Results showed that those genes relevant to risk score were mainly enriched in keratinocyte differentiation, and RHO GTPases activate PNKs, epithelial tube morphogenesis, establishment or maintenance of cell polarity, etc. Interestingly, we compared pathways predicted with annotations of these six RBPs in GeneCards^6^ and found that *HSPG2* acted as an anti-angiogenic and anti-tumor peptide that inhibited endothelial cell migration and collagen-induced endothelial tube morphogenesis. Angiogenesis is thought to be a critical procedure of promoting BLCA progression and associated with poor survival (Bochner et al., 1995; Roudnicky et al., 2017). Among these prognostic RBPs, *HSPG2* demonstrated an anti-angiogenesis effect via binding to α2β1 integrin and interacting with *VEGFR2* at the surface of endothelial cells (Woodall et al., 2008; Goyal et al., 2012; Poluzzi et al., 2016). A previous study has demonstrated that the breakdown of cell polarity programs could promote the occurrence of aggressive, invasive tumors (VanderVorst et al., 2018). In BLCA, *BMP4* could induce monocyte/macrophage polarization toward M2 phenotype macrophages, which promoted the progression of BLCA (Martinez et al., 2017). *FLNA* also regulated cell polarity by interacting with FilGAP, a Rac-specific GTPase-activating protein (Nakamura et al., 2009), but this mechanism had not been elucidated in BLCA. Exact mechanisms of these RBPs remain largely unknown and more research is required to investigate their roles in BLCA.

## Conclusion

In conclusion, we identified six RBPs associated with prognosis of BLCA and constructed the 6-RBP-based classifier to help clinical decision, while optimizing the predictive ability of the current TNM staging system. This study takes the initiative report that the RBP-based classifier could predict the prognosis in human BLCA. Nevertheless, large-scale, multi-center, and prospective studies are necessary to confirm our results before the 6-RBP-based signature can be applied in the clinic.

## Data Availability Statement

All datasets generated for this study are included in the article/supplementary material.

## Ethics Statement

This study is a secondary data analysis, and the raw data come from public databases. The institutional and/or national research ethics committee has approved the data collection and management process.

## Author Contributions

YW and YL: design, analysis, and interpretation of data, drafting of the manuscript, and critical revision of the manuscript. AH and BG: acquisition of data and statistical analysis. SH, CZ, and ZK: research direction. YG, XL, and LZ: critical revision of the manuscript for important intellectual content, administrative support, obtaining funding, and supervision. All authors contributed to the article and approved the submitted version.

## Conflict of Interest

The authors declare that the research was conducted in the absence of any commercial or financial relationships that could be construed as a potential conflict of interest.

## Footnotes

1 https://xena.ucsc.edu/

2 http://www.ncbi.nlm.nih.gov/geo/

3 http://metascape.org/

4 http://www.r-project.org/

5 https://www.proteinatlas.org/

6 https://www.genecards.org/

## Abbreviations

AUCs: Area under the curves
BLCA: Bladder cancer
circRNAs: Circular RNAs
DEGs: Differently expressed genes
DERBPs: DEGs coding for RNA-binding proteins
FD: Fold change
FDR: False discovery rate
GEO: Gene Expression Omnibus
HPA: Human Protein Atlas
HR: Hazard ratio
lncRNAs: Long non-coding RNAs
MIBC: Muscle-invasive bladder cancer
miRNAs: MicroRNAs
NMIBC: Non-muscle-invasive bladder cancer
OS: Overall survival
RBPs: RNA-binding proteins
RFS: Recurrence-free survival
ROC: Receiver operating characteristic
TCGA: The Cancer Genome Atlas
WGCNA: Weighted co-expression network analysis.
